# Beyond Triage: Cognitive Profiles and ED‐To‐Inpatient Costs and Resource Pathways in Older Adults

**DOI:** 10.1111/acem.70264

**Published:** 2026-03-22

**Authors:** Julia Biegelmeyer, Marlon J. R. Aliberti, Thiago J. Avelino‐Silva, Marcia M. P. Serra, Christian V. Morinaga, Pedro K. Curiati

**Affiliations:** ^1^ Geriatric Emergency Department Research Group (ProAGE), Hospital Sírio‐Libanês São Paulo São Paulo Brazil; ^2^ Geriatric Center for Advanced Medicine Hospital Sírio‐Libanês São Paulo Brazil; ^3^ Research and Education Institute Hospital Sírio‐Libanês São Paulo Brazil; ^4^ Division of Geriatrics University of California San Francisco San Francisco USA

## Abstract

**Background:**

Older adults are frequent users of the Emergency Department (ED), with a significant proportion presenting with pre‐existing or acute cognitive impairment. While negative post‐ED outcomes associated with cognitive status are well documented, their direct impact on care processes and resource allocation within the hospital remains poorly understood. This study aims to quantify how different cognitive profiles affect costs and care needs for acutely ill older adults.

**Methods:**

We conducted a secondary analysis of a prospective cohort study at a single, tertiary care hospital. We included patients aged ≥ 65 years admitted to the hospital through the ED. They were stratified into three groups based on the brief Confusion Assessment Method (bCAM) and the 10‐Point Cognitive Screener (10‐CS): normal cognition, cognitive impairment without delirium, and delirium. Primary outcome was cost of care. Resource utilization, characterized by the number of medical specialties involved, geriatric consultation, type of inpatient bed allocated from the ED, time to hospitalization, and patient satisfaction, were explored as secondary outcomes. Multiple regression models were used to assess associations, adjusting for sociodemographic factors, clinical severity, and geriatric vulnerability.

**Results:**

The sample comprised 824 patients: 429 (52.1%) with normal cognition, 165 (20.0%) with delirium, and 230 (27.9%) with cognitive impairment without delirium. Clinical severity, but not cognitive status, was independently associated with costs (B = 0.18; 95% CI: 0.08, 0.27). Delirium was independently associated with allocation to high‐complexity bed and receiving a geriatric consultation. Cognitive impairment was independently associated with a greater number of specialties involved.

**Conclusions:**

Clinical severity showed the strongest association with costs. In contrast, cognitive profiles were independently associated with the care pathway and complexity, with delirium linked to higher‐acuity allocation and preexisting cognitive impairment without delirium to broader multidisciplinary involvement. Recognizing these distinct cognitive profiles is fundamental for anticipating care demands and optimizing resource allocation for this vulnerable population.

## 1 Introduction

A global demographic shift towards an aging population is driving a significant and escalating demand on acute healthcare services, particularly the Emergency Department (ED) [1, 2, 3, 4, 5]. Brazil mirrors this global trend. Although the national health system comprises three distinct subsectors—public (government‐funded), private health insurance, and private out‐of‐pocket care [6]—the impact of population aging is consistent across all spheres. Older adults already account for a significant and growing proportion of ED visits and subsequent hospital admissions, with figures comparable to those observed in high‐income countries such as the United States [7, 8, 9]. This increased demand converges with systemic pressures, such as reduced inpatient bed capacity, culminating in ED overcrowding and prolonged boarding times [10, 11, 12]. Prolonged stays in the stimulating ED environment are an independent risk factor for in‐hospital mortality, adverse events, and subsequent functional decline [13, 14, 15].

Among the most vulnerable older adults in the ED are those with cognitive impairment. These patients face additional barriers to communicating their needs, making them highly susceptible to environmental risks and a vicious cycle of clinical deterioration. Cognitive impairment manifests primarily in two clinically distinct, yet often overlapping, profiles: chronic cognitive impairment, such as dementia and milder cognitive deficits, and acute delirium. While both conditions are associated with an increased risk of hospital admission, prolonged ED stays, adverse events, and mortality [16, 17, 18, 19, 20, 21, 22, 23, 24, 25], they present unique challenges for diagnosis and management. Delirium, as an acute medical emergency, requires immediate attention, yet its detection remains inconsistent in busy ED settings. Conversely, pre‐existing dementia is frequently under‐recognized [26, 27]. However, the impact of cognitive impairment on operational efficiency, resource allocation and quality of care remains poorly quantified. A recent scoping review by the Geriatric Emergency Care Applied Research 2.0—Alzheimer's Dementia Care (GEAR 2.0‐ADC) Network identified as a key research priority the investigation of “which environmental, operational, personnel, systemic, or policy changes improve care” for people with dementia [28].

This study aims to fill this gap by moving beyond a simple dementia vs. non‐dementia dichotomy. Leveraging data from a prospective cohort [29] that stratified older adults in the ED into three distinct cognitive profiles—normal cognition, delirium, and cognitive impairment without delirium—we analyzed the association of cognitive status with costs of care and a comprehensive set of operational, resource allocation, and quality of care outcomes.

## 2 Methods

### 2.1 Study Design and Setting

This was a secondary analysis of a prospective cohort study conducted at the Hospital Sírio‐Libanês (HSL), a private, tertiary‐care, internationally accredited teaching hospital in São Paulo, Brazil. The HSL ED manages over 80,000 visits annually and features a specialized Geriatric ED program (ProAGE), which has received the American College of Emergency Physician's (ACEP) Geriatric Emergency Department Accreditation (GEDA), dedicated to advancing health equity for older populations.

### 2.2 Study Population

We included patients aged 65 years or older who were admitted to the hospital from the ED. Exclusion criteria were: refusal to participate; absence of a reliable informant for patients with altered mental status; inability to be contacted for follow‐up interviews; and incomplete data in the utilized databases. To ensure independence of observations for the regression analyses, only the first eligible ED admission for each unique patient during the recruitment period was included in the study. All subsequent readmissions by the same individual were excluded.

### 2.3 Data Collection and Cognitive Assessment

Data were collected between November 2021 and April 2022. Trained research assistants conducted initial interviews after the decision to admit was made but before transferring to an inpatient bed. Sociodemographic and clinical data were collected and entered into a Research Electronic Data Capture (REDCap) database.

Cognitive status was determined using a structured, sequential two‐step process. First, delirium was screened for all patients using the brief Confusion Assessment Method (bCAM), which assesses acute onset and fluctuating course, inattention, disorganized thinking, and altered level of consciousness [30, 31]. The bCAM is a validated tool for identifying delirium in older ED patients, demonstrating high specificity (96.4%) and sensitivity (94%) in its Portuguese validation [31, 32, 33, 34]. Patients positive for bCAM were assigned to the “Delirium” group. Patients with a negative bCAM underwent further assessment with the 10‐Point Cognitive Screener (10‐CS), a validated tool that assesses temporal orientation, verbal fluency, and three‐word recall and takes approximately 2 min to administer, summarized in Box 1 and detailed in Figure S1 [35]. Importantly, the 10‐CS should not be administered in patients with delirium because this condition impairs attention and other cognitive functions, rendering dementia screening results uninterpretable. Based on established cutoffs adjusted for education, patients were classified as having preexisted cognitive impairment without delirium (bCAM negative and 10‐CS ≤ 5) or normal cognition (bCAM negative and 10‐CS ≥ 6).

BOX 1 **Assessment tools used in the study.**

**Table:** 

Purpose	Method	Interpretation
brief Confusion Assessment Method (bCAM)		
Rapid screening for delirium	Assesses four key features: (1) acute change or fluctuating course, (2) inattention, (3) disorganized thinking, (4) altered level of consciousness	A positive screen requires the presence of features 1 and 2, plus either 3 or 4
10‐point Cognitive Screener (10‐CS)		
Brief screening for pre‐existing cognitive impairment in patients without delirium	Assesses temporal orientation (month, day, year), category fluency (naming animals in 1 min), and three‐word recall. The score is adjusted for education level	A score of ≤ 5 is indicative of cognitive impairment. This cutoff was used to define the “Cognitive Impairment” group in this study
National Early Warning Score 2 (NEWS2)		
To quantify acute clinical severity and risk of physiological deterioration	Based on six physiological parameters: respiration rate, oxygen saturation, systolic blood pressure, pulse rate, level of consciousness, and temperature	Higher scores indicate greater physiological derangement, ranging from 0 to 20
PRO‐AGE Scoring system		
To assess geriatric vulnerability and predict the risk of adverse hospital outcomes	Evaluates six clinical variables: fatigue, age ≥ 90 years, recent hospitalization, weight loss, acute mental change, and acute functional decline	Higher scores indicate higher vulnerability, ranging from 0 to 8

This study was conducted in accordance with the principles of the Declaration of Helsinki and received full approval from the Research Ethics Committee at Hospital Sírio‐Libanês. All participants, or their legal proxies, provided written informed consent before their inclusion in the study.

### 2.4 Outcomes and Covariates

The primary outcome was the cost of care, which represents the total hospital billed amount, including the cost of the hospital stay and fees, medications administered, consumables, high‐cost materials and medications, diagnostic tests and procedures. Medical fees were not included. Admission costs were standardized by converting individual values to z‐scores using the mean and standard deviation of all hospital admissions during the study period. Standardization was performed solely to rescale the variable and facilitate comparability across measures with different units and magnitudes. No assumptions of normality were made, and the standardized values were not interpreted probabilistically, but rather as relative positions within the study population.

The selection of secondary outcomes was designed to provide a multifaceted assessment of care, encompassing standard health services metrics as well as more granular process‐of‐care indicators. We analyzed as secondary outcomes: (1) allocation from the ED to a high‐complexity bed (monitored unit or intensive care unit, ICU) versus a standard ward bed; (2) time from ED admission to hospitalization, defined as the elapsed time between the initial physician evaluation in the emergency department and the moment when hospital admission was formally recorded in the institutional information system. These are widely validated and generalizable metrics used to evaluate operational efficiency and resource consumption in acute care settings [14, 36]. To provide a deeper understanding of the care process itself, we also included two novel metrics: (3) number of medical specialties involved in care during the hospital stay, as a proxy for care fragmentation; (4) the request for a geriatric consultation during the hospital stay, as an indicator of specialized care recognition. Finally, to analyze quality of care, we included: (5) patient satisfaction, using the Net Promoter Score (NPS) [37]. Originally developed and validated in the business sector as a metric for customer loyalty and experience, the NPS has been increasingly adopted and validated within healthcare settings as a pragmatic, standardized tool to assess the patient experience [38, 39]. NPS consists of a simple, two‐part questionnaire. Firstly, respondents answer the question: “How likely is it that you would recommend our business/service to a friend or colleague?” on a scale from 0 (not likely) to 10 (very likely). Secondly, the respondents answer a free‐text item explaining the main reason for their score. Responses are categorized into three groups: “Detractors” (ratings 0–6), “Passives” (ratings 7 and 8) and “Promoters” (ratings 9 and 10) [40]. This questionnaire was sent to the patient or the responsible caregiver by e‐mail 12 h after discharge. This approach aligns with the growing emphasis on patient‐centered outcomes in geriatric emergency departments, which prioritize the patient's experience alongside clinical and operational metrics [41].

All data were extracted from the hospital's electronic medical record system (Philips Tasy EMR).

Covariates for adjustment in the multivariable models were selected a priori based on established evidence. They included sociodemographic characteristics (age and sex, as fundamental demographic confounders in geriatric research [25], and marital status, as a proxy for social support, a known determinant of healthcare utilization and outcomes in older adults [42, 43]) and two key constructs: clinical severity and geriatric vulnerability.

Clinical severity was assessed using National Early Warning Score 2 (NEWS 2), a validated tool that measures illness acuity, comprising seven physiological variables that often integrate early warning systems to identify high‐risk patients in acute care settings, and has good prediction of 30‐day mortality [44]. This tool allows the health team to early recognize when the escalation of care to a critical care team is appropriate. NEWS was designed to be generic and should reflect the physiological perturbations associated with various comorbidities [44].

For the multifaceted construct of geriatric vulnerability—which encompasses frailty, multimorbidity, and functional status—we selected the PRO‐AGE scoring system. This choice was deliberate for several reasons. First, the PRO‐AGE scoring system was specifically developed and validated in the ED environment and has demonstrated superior or comparable predictive accuracy for key hospital outcomes (hospital admission, prolonged stays, in‐hospital mortality and activities of daily living disability) when compared to other instruments like the ISAR or FRAIL scale in this specific clinical context [45, 46]. Second, the PRO‐AGE scoring system is a composite tool that captures multiple geriatric domains simultaneously, integrating not only chronic disease burden (via hospitalization history) but also sociodemographic characteristics (older age) and core geriatric syndromes like functional decline, exhaustion, weight loss, and acute mental status change, which are often more predictive of outcomes in older adults than a simple count of diseases [47]. Critically, to prevent collinearity with our primary exposure variable (cognitive status), both the NEWS2 and PRO‐AGE scores were modified by removing their respective items related to “acute mental status change”. While methodologically necessary, we acknowledge that this modification deviates from the originally validated versions of the score. However, we argue that the remaining components of the PRO‐AGE score (age, recent hospitalization, weight loss, exhaustion, functional decline) continue to provide a robust and clinically relevant measure of baseline geriatric vulnerability, independent of the acute cognitive insult. This approach allowed us to isolate cognitive status while still robustly adjusting for both the patient's acute physiological derangement and their underlying vulnerability. In addition, a separate study validated a modified version of the PRO‐AGE scoring system in hospitalized COVID‐19 older patients, demonstrating that it remained a robust predictor of mortality compared with other prognostic tools, even when controlled for the Charlson Comorbidity Index (CCI), age, sex and illness acuity [48].

Detailed operational definitions and scoring instructions for the screening instruments used in this study are provided in Tables S1 and S2 and summarized in Box 1.

### 2.5 Statistical Analysis

Analyses were performed using SPSS version 22.0. As quantitative variables were non‐normally distributed (Shapiro–Wilk test), they are presented as medians and interquartile ranges (IQR). Categorical variables are presented as frequencies and percentages. Comparisons between the three cognitive groups were made using the Kruskal‐Wallis test for continuous variables and the Chi‐Square or Fisher's Exact test for categorical variables.

Multiple linear regression was used for continuous outcomes (cost, number of specialties, time, and NPS), while binary logistic regression was used for dichotomous outcomes (geriatric assessment, bed type). All models were adjusted for pre‐specified covariates and fitted using a direct entry approach. To ensure consistent adjustment across analyses and to avoid biases associated with automated variable selection procedures, all covariates selected a priori were retained in the final models regardless of statistical significance.

Sample size was determined a priori to ensure adequate statistical power (80%) to detect a medium effect size at a significance level of *α* = 0.05. Based on these assumptions, the minimum required sample was 111 participants for the linear regression models and 175 for the logistic regression models. The final sample size of the study was deemed sufficient to perform all planned multivariable analyses [49, 50]. All tests were two‐tailed.

Although our quantitative outcome variables were not normally distributed, standard multiple linear regression was deemed appropriate for the analysis due to its robustness in large samples, where the Central Limit Theorem supports the validity of statistical inferences [49, 51]. Admission costs were standardized using z‐scores for scaling purposes, and given the skewed distribution of this variable, medians are reported throughout the text.

Prior to the final regression analysis, diagnostic procedures were performed to evaluate model assumptions and validity. Multicollinearity among independent variables was evaluated using the Variance Inflation Factor (VIF) and tolerance statistics across all models. For the linear regression models, residuals were inspected for homoscedasticity and influential outliers. For logistic regression models, overall goodness‐of‐fit was assessed using the Hosmer‐Lemeshow test.

Model performance was assessed using the F‐statistic and *R* ^2^ for linear regressions, and the Omnibus Test and Nagelkerke *R* ^2^ for logistic regressions. While the *R* ^2^ and pseudo‐*R* ^2^ values were modest, as expected in observational studies of complex clinical phenomena, all primary models demonstrated statistically significant overall fit, supporting the interpretation of independent associations between covariates and outcomes.

Missing data were handled using a complete case analysis approach; individuals with missing data for any variables included in a given regression model were excluded from that analysis.

## 3 Results

**FIGURE 1:**
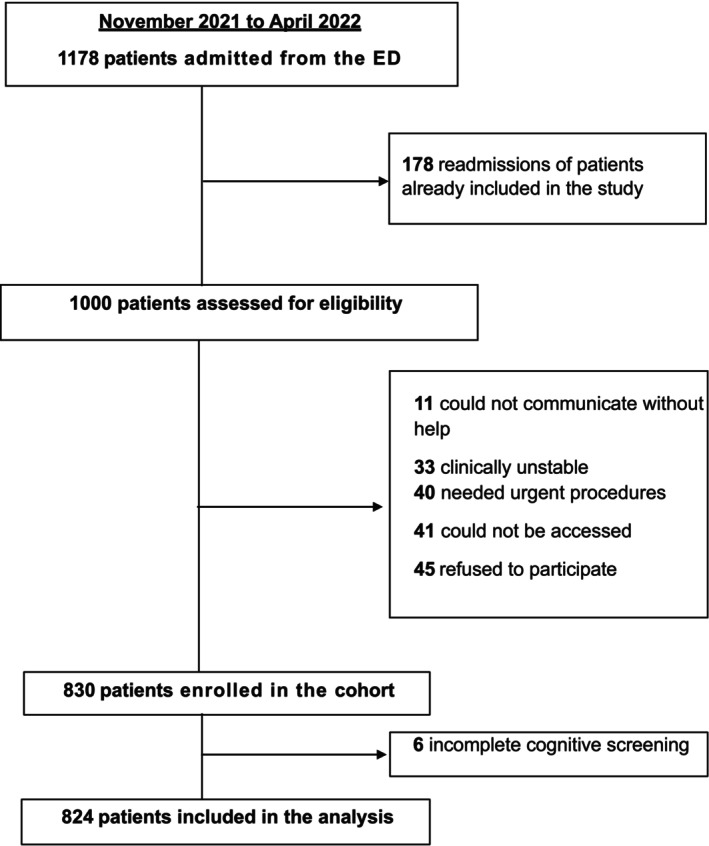
Flowchart of the study participants.

Of 1178 admissions during the study period, 830 patients were included in the cohort (Figure 1). After excluding six patients with incomplete cognitive screening data, the final analysis included 824 patients. The sample was stratified into three cognitive groups: normal cognition (*n* = 429, 52.1%), cognitive impairment without delirium (*n* = 230, 27.9%), and delirium (*n* = 165, 20.0%).

### 3.1 Baseline Characteristics

As shown in Table 1, significant differences were observed across the three groups. The median age of the sample was 79 (IIQ 13) and over half were males (53.5%). Patients in the Delirium group were older, had the lowest levels of education, and the highest proportion of unmarried/widowed patients and residents of long‐term care facilities. Furthermore, they were more disabled, multimorbid, frail and were more frequently prescribed with anticholinergic medication. Consequently, this group also had the highest scores for clinical severity (modified NEWS2) and geriatric vulnerability (modified PRO‐AGE). Dementia group showed intermediate results. These significant baseline differences underscore the necessity of multivariable adjustment to isolate the independent effect of cognitive status on outcomes.

**TABLE 1:** Sociodemographic and clinical characteristics of the study population and outcomes.

Characteristic	Total sample (*n* = 824)	Normal cognition (*n* = 429)	Cognitive impairment (*n* = 230)	Delirium (*n* = 165)	*p*
Sociodemographic characteristics					
Age (years), median (IQR)	79 (13)	75 (10)	82 (12)	88 (11)	**< 0.001**
Male sex, *n* (%)	441 (53.5)	252 (58.7)	113 (49.1)	76 (46.1)	**0.006**
Education (years), median (IQR)	15 (6)	16 (6)	14 (6)	11 (5)	**< 0.001**
Ethnicity, *n* (%)					
White	759 (92.1)	397 (92.5)	210 (91.3)	152 (92.1)	0.367
Black	11 (1.3)	6 (1.4)	4 (1.7)	1 (0.6)	
Mixed‐race (Parda)	29 (3.5)	18 (4.2)	7 (3)	4 (2.4)	
Asian (Amarela)	25 (3)	8 (1.9)	9 (3.9)	8 (4.8)	
Marital Status, *n* (%)					
Partnered	466 (56.6)	276 (64.3)	115 (50)	75 (45.5)	**< 0.001**
Not partnered	358 (43.4)	153 (35.7)	115 (50)	90 (54.5)	
Living Arrangement, *n* (%)					
With family	616 (74.8)	333 (77.6)	170 (73.9)	113 (68.5)	**< 0.001**
Long‐term care facility	10 (1.2)	0 (0)	2 (0.9)	8 (4.8)	
Alone	198 (24)	96 (22.4)	58 (25.2)	44 (26.7)	
Needs help with self‐care, *n* (%)	276 (33.5)	37 (8.6)	84 (36.7)	155 (93.9)	**< 0.001**
Clinical characteristics					
Comorbidities, *n* (%)					
Stroke or TIA	93 (11.3)	39 (9.1)	23 (10)	31 (18.8)	**0.003**
Parkinson's disease	44 (5.3)	10 (2.3)	10 (4.3)	24 (14.5)	**< 0.001**
Depression	147 (17.8)	68 (15.9)	31 (13.5)	48 (29.1)	**< 0.001**
Dementia	156 (18.9)	13 (3)	30 (13)	113 (68.5)	**< 0.001**
CCI, median (IQR)	1 (2)	1 (2)	1 (3)	2 (2)	**0.002**
Poor vision, *n* (%)	57 (6.9)	18 (4.2)	13 (5.7)	26 (15.8)	**< 0.001**
Poor hearing, *n* (%)	167 (20.3)	58 (13.5)	51 (22.2)	58 (35.2)	**< 0.001**
ED visit in last 6 months, *n* (%)	336 (40.8)	157 (36.6)	87 (37.8)	92 (55.8)	**< 0.001**
Hospitalization > 24 h in last 6 months, *n* (%)	339 (41.1)	151 (35.2)	97 (42.2)	91 (55.2)	**< 0.001**
Katz Index, median (IQR)	0 (3)	0 (0)	1 (3)	5 (2.5)	**< 0.001**
Frailty (CFS ≥ 5), *n* (%)	224 (27.2)	30 (7)	59 (25.7)	135 (81.8)	**< 0.001**
Polypharmacy, *n* (%)	561 (68)	240 (56)	178 (77.4)	143 (86.6)	**< 0.001**
Anticholinergic medication use, *n* (%)	159 (19.3)	58 (13.5)	44 (19.1)	57 (34.5)	**< 0.001**
PIM use, *n* (%)	714 (86.7)	345 (80.4)	214 (93)	155 (93.9)	**< 0.001**
NEWS2, median (IQR)	1 (3)	1 (2)	1 (2)	2 (3)	**< 0.001**
PRO‐AGE, median (IQR)	2 (2)	1 (2)	2 (3)	4 (2)	**< 0.001**
Outcomes					
Cost, in Z‐scores, median (IQR)	0.46 (1.07)	0.30 (0.82)	0.48 (1.06)	0.98 (1.41)	**< 0.001**
Bed type, *n* (%)					
Ward or step‐down unit	593 (72)	333 (77.6)	174 (75.6)	86 (52.1)	**< 0.001**
Monitored or ICU	231 (28)	96 (22.4)	56 (24.4)	79 (47.9)	
Geriatric assessment, *n* (%)	254 (33)	85 (21.3)	82 (38.7)	87 (55.4)	**< 0.001**
Number of medical specialties, median (IQR)	5 (4)	4 (3)	5 (3)	6 (4)	**< 0.001**
Time to inpatient admission (hours), median (IQR)	1.22 (1.24)	1.31 (1.36)	1.25 (1.24)	1.08 (1.01)	**0.021**
NPS, median (IQR)	10 (0)	10 (0.25)	10 (0)	10 (0.75)	0.792

*Note:* Bold values indicates all *p* values (statistical significance).

Abbreviations: CCI, Charlson Comorbidity Index; CFS, Clinical Frailty Scale; ED, emergency department; ICU, intensive care unit; IQR, interquartile range; NEWS2, National Early Warning Score 2; NPS, Net Promoter Score; PIM, Potentially Inappropriate Medication; TIA, transient ischemic attack.

### 3.2 Multivariable Regression Analysis

**FIGURE 2:**
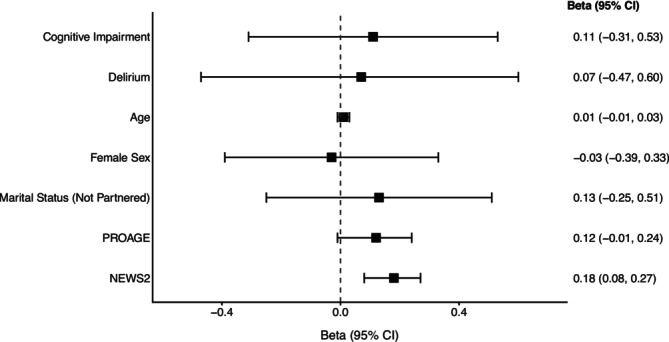
Association between cognitive status and covariates with care costs.

After adjusting for covariates, cognitive status was no longer associated with the cost of care. The only variable that remained associated with higher costs was clinical severity, as measured by the modified NEWS2 score (B = 0.18; 95% CI: 0.08, 0.27), as shown in Figure 2.

Further results of the adjusted regression analyses are presented in Table 2. Delirium remained independently associated with a higher likelihood of allocation to a monitored or ICU bed (adjusted odds ratio (aOR) 2.61; 95% CI: 1.60, 4.27) and receiving a geriatric consultation (aOR 2.07; 95% CI: 1.26, 3.40).

**TABLE 2:** Multivariate regressions exploring the association between cognitive status and ED outcomes.

	Time to admission (*n* = 817)	Number of specialties (*n* = 769)	NPS score (*n* = 124)	Allocation to monitored bed (*n* = 824)	Geriatric consultation (*n* = 769)
	B (95% CI)	B (95% CI)	B (95% CI)	aOR (95% CI)	aOR (95% CI)
Cognitive impairment	−0.10 (−0.36, 0.15)	0.72 (0.20, 1.23)	−0.38 (−1.00, 0.24)	1.03 (0.68, 1.55)	**1.52 (1.02, 2.28)**
Delirium	−0.23 (−0.57, 0.10)	0.50 (−0.16, 1.17)	−0.05 (−1.01, 0.92)	**2.61 (1.60, 4.27)**	**2.07 (1.26, 3.40)**

*Note:* All analyses were adjusted for sociodemographic characteristics (age, sex, marital status), clinical severity (NEWS2), and geriatric vulnerability (PRO‐AGE). Bold values indicates significance values in the form of 95% CI

Abbreviations: aOR, adjusted odds ratio; CI, confidence interval; NPS, Net Promoter Score.

The presence of cognitive impairment without delirium was independently associated with an increase in the number of medical specialties involved in care (B = 0.72; 95% CI: 0.20, 1.23) and a higher likelihood of geriatric consultation (aOR 1.52; 95% CI: 1.02, 2.28).

## 4 Discussion

In this study of 824 acutely ill older adults admitted through the emergency department, we identified a dissociation between the factors driving hospital costs and those shaping care complexity. Acute physiological severity emerged as the sole independent determinant of initial hospitalization costs, whereas patients' cognitive profiles were independently associated with downstream care pathways. Specifically, delirium was associated with a higher likelihood of allocation to high‐acuity beds, while pre‐existing cognitive impairment was linked to greater fragmentation of care across multiple medical specialties.

A key strength of this study lies in the stratification of patients into three clinically distinct cognitive groups. Prior studies have often treated delirium and dementia as overlapping or interchangeable risk factors, potentially obscuring their distinct clinical implications. By disentangling acute confusional states from chronic cognitive impairment, our analysis aligns with evidence showing that delirium superimposed on dementia follows a different prognostic trajectory than either condition alone [52, 53, 54]. This approach allowed us to demonstrate that delirium primarily influences the intensity of care required, whereas chronic cognitive impairment predominantly affects care coordination demands.

Another methodological strength is our use of the PRO‐AGE score to adjust for baseline geriatric vulnerability. Unlike traditional comorbidity indices like the CCI, which may not fully capture multidimensional risk in older adults, PRO‐AGE incorporates functional, clinical, and recent health trajectory domains and has been validated in ED settings [45, 46, 48]. This comprehensive adjustment strengthens our finding that immediate hospitalization costs were driven by acute illness severity rather than baseline vulnerability or cognitive status.

Our finding that clinical severity was the primary driver of costs contrasts with population‐level studies where dementia [36, 55] and delirium [56] are associated with higher total hospitalization costs. This apparent discrepancy suggests that the economic burden associated with cognitive impairment may arise predominantly from downstream factors—such as prolonged length of stay, increased complication rates, and higher care needs—rather than from the initial diagnostic and therapeutic processes in the ED. This interpretation is consistent with prior work demonstrating increased preventable adverse events, including infections and falls, among hospitalized patients with dementia, as demonstrated by Bail et al. [57].

The independent association of delirium with ICU/monitored bed allocation, even after adjusting for NEWS2, is a novel finding. It suggests that clinicians may perceive delirium as a marker of acute organ dysfunction and clinical instability that warrants higher‐intensity monitoring beyond what is captured by vital sign–based severity scores alone. This finding challenges the notion that age or baseline dependency are primary barriers to critical care access, as suggested by Foley et al. [58], and instead highlights delirium itself as a key decision‐making factor.

Conversely, the association between chronic cognitive impairment and the involvement of multiple medical specialties may reflect a pattern of diagnostic and therapeutic uncertainty. Cognitive impairment can complicate history‐taking, symptom interpretation, and decision‐making, potentially prompting broader consultation as clinicians attempt to clarify complex presentations and balance competing risks. This fragmentation of care underscores the need for structured geriatric approaches to improve coordination and efficiency in this vulnerable population.

Finally, patient satisfaction scores were uniformly high across cognitive groups, despite marked differences in care pathways. These findings must be interpreted cautiously due to substantial selection bias; however, they are consistent with qualitative evidence suggesting that older adults often prioritize interpersonal aspects of care—such as communication, respect, and symptom control—over operational characteristics when evaluating their emergency care experience [59].

## 5 Limitations

This study has limitations. First, its observational design precludes inferences of causality. Second, external validity is limited by the single‐center setting. The research was conducted in a high‐complexity private hospital in Brazil, serving a patient population with a socioeconomic and educational profile that does not reflect the broader Brazilian population. Brazil's healthcare system is a fragmented public–private structure, with the majority of the population relying exclusively on the publicly funded Unified Health System (SUS), which operates under vastly different resource constraints, patient demographics, and operational pressures [6, 60, 61]. Therefore, our findings, while internally valid, may not be directly generalizable to public hospitals in Brazil or to lower‐resource settings. However, the characteristics of our institution—a resource‐rich, high‐acuity center with ready access to specialized services—may enhance the relevance of our findings to similar private or academic medical centers in countries with mixed healthcare systems, including parts of North America and Europe.

Third, the method for counting medical specialties may have overestimated the true number if a single specialist with multiple board certifications made entries. Fourth, the analysis of patient satisfaction was severely limited by a very low response rate (15%), leading to a high risk of selection bias and insufficient statistical power; these results should be interpreted with extreme caution.

Finally, our study analyzed six distinct outcomes using separate regression models without applying a formal correction for multiple comparisons. Therefore, we cannot exclude the possibility that some of the statistically significant associations occurred by chance. The results should, therefore, be interpreted with this consideration in mind. Our rationale for this approach was twofold. First, this is an observational, hypothesis‐generating study where each outcome represents a pre‐specified, distinct clinical and operational question. Second, adjustments for multiple comparisons are often conservative and can substantially increase the risk of Type II errors, potentially obscuring clinically meaningful associations that warrant further investigation.

## 6 Conclusions

Cognitive status is a potential driver of care needs and pathways for older adults in the ED. Delirium signals a need for high‐intensity monitoring and specialized geriatric expertise, while chronic cognitive impairment signals a need for enhanced care coordination to prevent fragmentation. While clinical severity is the primary driver of costs, proactive identification and differentiated management of these distinct cognitive profiles in the ED are therefore fundamental for anticipating care demands, ensuring patient safety, and enabling the efficient allocation of healthcare resources.

## Author Contributions

J.B.: study concept and design, acquisition of data, analysis and interpretation of data, and drafting of the manuscript. M.J.R.A.: study concept and design, drafting and revision of the manuscript. T.J.A.‐S.: drafting and revision of the manuscript. M.M.P.S.: analysis of data. C.V.M.: acquisition of data, analysis, and interpretation of data. P.K.C.: study concept and design, acquisition of data, analysis and interpretation of data, and drafting of the manuscript.

## Funding

This study was funded by the Geriatrics Research Nucleus of the Hospital Sírio‐Libanês.

## Conflicts of Interest

The authors declare no conflicts of interest.

## Supporting information


**Data S1:** acem70264‐sup‐0001‐DataS1.docx.

## Data Availability

The data that support the findings of this study are available on request from the corresponding author. The data are not publicly available due to privacy or ethical restrictions.
