# Supplementary material for: Beyond Triage: Cognitive Profiles and ED‐To‐Inpatient Costs and Resource Pathways in Older Adults
Source: Acad Emerg Med. 2026 Mar 22;33(3):e70264. doi: 10.1111/acem.70264 (PMC13006781; doi:10.1111/acem.70264)
Supplement: Supplementary file 1 — Data S1: acem70264‐sup‐0001‐DataS1.docx. [file ACEM-33-0-s001.docx]

**Supplemental material**

This supplemental material is for online-only contains:

Figure S1: 10-Cognitive Screener

Table S1**:** National Early Warning Score (NEWS) 2

Table S2. PRO-AGE scoring system

**Figure S1: 10-Cognitive Screener**

- WHAT YEAR IS THIS? 0 1
- WHAT MONTH IS THIS? 0 1
- WHAT IS TODAY’S DATE? 0 1

**Now I am going to name three objects. Please wait until I say all three words, then repeat them. Remember what they are. I am going to ask you to name them again in a few minutes. Please repeat these words for me:**

CAR – VASE – BRICK

(may repeat 3 times if necessary; repetition is not scored)

- **Now tell me the names of all animals you can think of, as quick as possible. I’ll give you one minute to come up with as many animals as you can. Ready?**

0–5: 0

6–8: 1

9–11: 2

12–14: 3

15 or more: 4

- **Now what were the three objects I asked you to remember?**

CAR 0 1

VASE 0 1

BRICK 0 1

10-CS: _____

Adjustment for Education Effects:

- No formal education: add 2 points (to a max of 10)
- 1–3 years of education: add 1 point (to a max of 10)

10-CS-Edu: _____

Interpretation of the 10-CS-Edu:

- ≥ 8 points: Normal
- 6–7 points: Possible cognitive impairment
- 0–5 points: Probable cognitive impairment

Adapted from: Apolinario D, Lichtenthaler DG, Magaldi RM, Soares AT, Busse AL, Amaral JR, et al. Using temporal orientation, category fluency, and word recall for detecting cognitive impairment: the 10-point cognitive screener (10-CS). Int J Geriatr Psychiatry. 2016;3

**Table S1 –** National Early Warning Score (NEWS) 2

| **The National Early Warning Score 2 (NEWS 2; 0-20)^a^** | | | | |
| --- | --- | --- | --- | --- |
| ***Physiological parameters*** | **0** | **+1** | **+2** | **+3** |
| Respiration rate (breaths per min) | 12-20 | 9-11 | 21-24 | ≤ 8  ≥ 25 |
| Blood oxygen saturation levels (SpO_2_)  Scale 1 | 96-100 | 94-95 | 92-93 | ≤ 91 |
| Blood oxygen saturation levels (SpO_2_) Scale 2^b^ | 88–92  ≥93 on air | 86–87  93–94 on  oxygen | 84–85  95–96 on  oxygen | ≤83  ≥97 on  oxygen |
| Any supplemental oxygen? | No |  | Yes |  |
| Temperature (°C) | 36.1-38.0 | 35.1-36.0 or  38.1-39.0 | ≥ 39.1 | ≤ 35.0 |
| Systolic blood pressure (mmHg) | 111-219 | 101-110 | 91-100 | ≤ 90 or  ≥ 220 |
| Heart/pulse rate (beats per minute) | 51-90 | 41-50 or  91-110 | 111-130 | ≤ 40 or  ≥ 131 |
| Altered level of consciousness (AVPU)^c^ | Alert |  |  | Voice, Pain, Unresponsive |
| Thresholds and triggers: Score from 0-4 is considered low risk and requires ward-based response; score =3 in any individual parameter is considered low-medium risk and requires urgent ward-based response; Score 5-6 corresponds to medium risk and is a key threshold for urgent response; score 7 or more is considered high risk and triggers urgent or emergency response | | | | |

^a^ *In our study, we excluded the AVPU item to avoid collinearity with delirium group.*

*^b^ The usual scale is number 1; Scale 2 is blood oxygen saturation levels specific to patients with hypercapnic respiratory failure (usually COPD) who require their ‘usual’ oxygen saturations to be set at 88–92%.*

*^c^ We calculated the AVPU (Alert, Voice, Pain, Unresponsive) scale equivalent using the three domains of the Glasgow Coma Scale (eye-opening, verbal response, and motor response), adopting a method from previous work (Usman OA, et al. Comparison of SIRS, qSOFA, and NEWS for the early identification of sepsis in the Emergency Department. Am J Emerg Med. 2019;37(8):1490-1497).*

*Adapted from: Royal College of Physicians. National Early Warning Score (NEWS) 2: Standardising the assessment of acute-illness severity in the NHS. Updated report of a working party. London: RCP, 2017.*

**Table S2**. PRO-AGE scoring system

| **PRO-AGE score (0-8) ^a^** | | |
| --- | --- | --- |
| ***Items*** | ***Points*** | |
| **Physical impairment**: "Since the illness that brought you to the Emergency Department, have you needed more help than usual to take care of yourself?" | No = 0 | Yes = 2 |
| **Recent hospitalization**: "Have you been hospitalized for one or more nights during the last six months?" | No = 0 | Yes = 1 |
| **Older age**: "Is the patient's age 90 years or older?" ^a^ | No = 0 | Yes = 1 |
| **Acute mental alteration**: "Is there evidence of an acute change in mental status from the patient's baseline?" | No = 0 | Yes = 2 |
| **Getting thinner**: "Have you had a weight loss of 5% or more during the last year?" | No = 0 | Yes = 1 |
| **Exhaustion**: "How much time during the previous 4 weeks did you feel tired?"  1 = All the time, 2 = Most of the time, 3 = Some of the time, 4 = A little of the time, 5 = None of the time. | 3 to 5 = 0 | 1 or 2 = 1 |

*^a^ In our study, we divided our sample according to cognitive status, therefore, we excluded “Acute mental alteration” from the algorithm to avoid collinearity, which resulted in scores ranging from 0 to 6 (higher = worse).*

*^b^ Refers to the period immediately before the symptoms of acute illness have started ("baseline exhaustion").*

*Adapted from: Curiati PK, Gil-Junior LA, Morinaga C V., Ganem F, Curiati JAE, Avelino-Silva TJ. Predicting Hospital Admission and Prolonged Length of Stay in Older Adults in the Emergency Department: The PRO-AGE Scoring System. Ann Emerg Med. 2020 Sep;76(3):255–65. doi:10.1016/j.annemergmed.2020.01.010*
